# Polymer–carbon dot hybrid structure for a self-rectifying memory device by energy level offset and doping

**DOI:** 10.1039/c8ra01928b

**Published:** 2018-04-16

**Authors:** Hang Lu, Yingying Chen, Qing Chang, Shuai Cheng, Yamei Ding, Jie Chen, Fei Xiu, Xiangjing Wang, Chaoyi Ban, Zhengdong Liu, Juqing Liu, Wei Huang

**Affiliations:** Key Laboratory of Flexible Electronics (KLOFE), Institute of Advanced Materials (IAM), Jiangsu National Synergistic Innovation Center for Advanced Materials (SICAM), Nanjing Tech University (NanjingTech) 30 South Puzhu Road Nanjing 211816 P. R. China iamjqliu@njtech.edu.cn iamwhuang@njtech.edu.cn

## Abstract

A strategy for self-rectifying memory diodes based on a polymer–carbon dot hybrid structure, with a configuration of rGO/PEDOT : PSS/carbon dots/MEH-PPV/Al, has been proposed. The fabricated device exhibits a rectification of 10^3^ in the rectification model and an ON/OFF current ratio of 121 in the memory model. The rectifying behavior was attributed to an energy level offset between the electrodes and the bilayer polymers and the memory effect was induced by carrier trapping of carbon dots within the polymers.

## Abstract 2

A strategy for self-rectifying memory diodes based on a polymer–carbon dot hybrid structure, with a configuration of rGO/PEDOT : PSS/carbon dots/MEH-PPV/Al, has been proposed.

## Graphical abstract

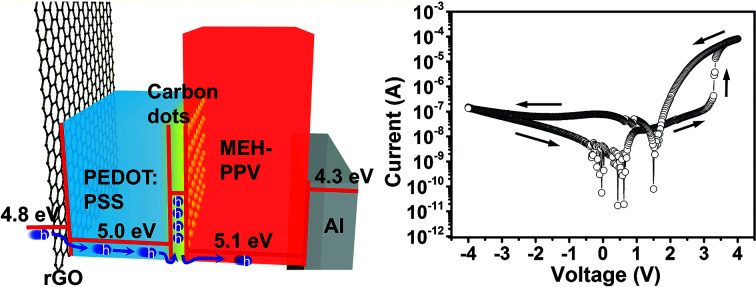

Polymer-based resistive switching memory devices, as ideal candidates for future emerging memory devices, have attracted a great deal of attention due to their simple structure, tunable properties, high-density integration, low-power consumption, facile fabrication process, and low-cost potential.^1–4^ Generally, a cross-bar architecture in memory arrays has been designed to achieve high-density data storage.^5–8^ However, the sneaking current issue, which is caused by a cross-talk effect in the cross-bar array, results in a misreading of a cell in a high resistance state (HRS) when the neighboring cells are in a low resistance state (LRS).^9–13^ To alleviate the sneaking current issue, great effort has therefore been devoted to the search for a memory device with a rectifying effect. For example, the architecture of one diode-one resistor (1D1R) or one transistor-one resistor (1T1R) can improve reading accessibility in an integrated memory array structure.^11,12,14,15^ However, they still suffer from limitations of complex device structure, fabrication process, low yield and high energy consumption,^16^ which could be circumvented by creating a self-rectifying memory device with a simple sandwich architecture and solution process fabrication.

Self-rectifying memory devices with metal/insulator/metal structure have risen as an important class of memory technology in high density data storage. Numerous transition metal oxide materials, *e.g.*, Cr-doped SrTiO_3_,^16^ Pr_0.7_Ca_0.3_MnO_3_ (PCMO),^17^ ZrO_2_,^18^ TiO_2_,^19^ HfO_2−*X*_,^20,21^ and TaO_*X*_,^22^ and Si-based materials, *e.g.*, a-Si^23,24^ and Si_3_N_4_,^25^ can serve as the insulator layer and exhibit excellent self-rectifying memory features such as short switching time, large resistance ratio, and good retention ability. Unfortunately, most devices are fabricated by traditional film plating technology such as pulsed laser deposition, electron beam deposition, sputtering deposition and thermal evaporation, leading to very complicated process. Nowadays, polymer rectifying devices by energy level offset^26,27^ and memory devices by doping method^28,29^ have been widely fabricated, with the merit of solution process. Therefore, combining energy level offset and doping technique in solution processed polymer diodes are anticipated to achieve self-rectifying memory performance.

In this letter, we reported a solution processed polymer–carbon dots hybrid structure for self-rectifying memory device by energy level offset and doping method, with a configuration of reduced graphene oxide (rGO)/poly(3,4-ethylenedioxythiophene) : poly(styrenesulfonate) (PEDOT : PSS)/carbon dots/poly(2-methoxy-5(2′-ethyl)hexyloxy-phenylenevinylene) (MEH-PPV)/Al. Bilayer PEDOT : PSS and MEH-PPV are used as the rectifying active layers due to their energy level offset with electrodes. Carbon dots are doped as the memory active layer due to their carrier trapping behavior within polymers. rGO film as bottom electrode and Al as top electrode are fabricated by thermal annealing and thermal evaporation, respectively. The fabricated device in a 6 × 6 cross-bar array exhibits rectifying function with a rectifying ratio of 10^3^, and also possesses stable memory effect with a minimum ON/OFF current ratio of 121. Our strategy is promising for preparation of other polymer–nanoparticle hybrid structures for self-rectifying memory devices.

The self-rectifying memory devices were fabricated basing on a facial solution-based process as shown in Fig. 1. The patterned rGO electrodes with a square resistance of 1 kΩ sq^−1^ were fabricated from solution-processed GO films *via* an oxygen-plasma etching approach.^30,31^ A 30 nm-thick PEDOT : PSS layer was spin-coated on rGO electrode surface, then treated with 20 W oxygen-plasma for 20 s, followed by thermal annealing in air at 120 °C for 20 min. The carbon dots aqueous with a concentration of 3 mg ml^−1^ was spin-coated onto PEDOT : PSS layer at 3000 rpm and subsequently annealed at 100 °C for 20 min. Subsequently, a 30 nm-thick MEH-PPV layer was spin-coated and then annealed in a N_2_ gas environment at 70 °C for 30 min. Finally, 6 Al lines of 500 μm in width were deposited perpendicularly to rGO lines, through a shadow mask *via* thermal evaporation. Electrical properties of the as-fabricated devices were investigated using a semiconductor parameter analyzer (Keithley 4200) in the ambient environment.

**Fig. 1:**
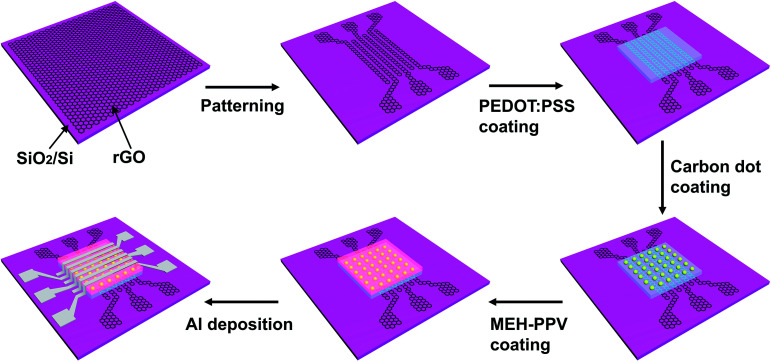
Schematic diagrams of the fabrication process for rGO/PEDOT : PSS/carbon dots/MEH-PPV/Al self-rectifying memory devices.

To illuminate that the memory effect is caused by the carrier trapping effect of carbon dots, the rectifying device without carbon dots was fabricated and characterized (Fig. 2(a)). The current–voltage (*I*–*V*) characteristics of the rectifying diode device are shown in Fig. 2(b). Basing on optimal fabrication conditions, the rectifying diode device with the optimized structure exhibited a maximum rectification ratio (RR) of ∼10^3^ following RR = |*I*_forward_/*I*_reverse_ (here – *I*(*V*−)/*I*(*V*+))|.^32^ This rectifying effect is attributed to the Schottky barrier between Al electrode and MEH-PPV layer when a negative bias is applied to the rGO electrode. As is shown in the energy band diagram (Fig. 2(c)), the work function of rGO and Al is 4.8 eV and 4.3 eV, respectively. The highest occupied molecular orbital (HOMO) of PEDOT : PSS and MEH-PPV occurs at 5.2 and 5.1 eV, respectively. In this case, during the positive voltage sweep, holes from the rGO electrode can be efficiently injected into the HOMO of PEDOT : PSS layer with a 0.4 eV barrier height. On the other hand, when applying a negative voltage, it's difficult to realize the injection of holes from Al electrode to the LUMO of MEH-PPV layer due to a large barrier height up to 0.8 eV.

**Fig. 2:**
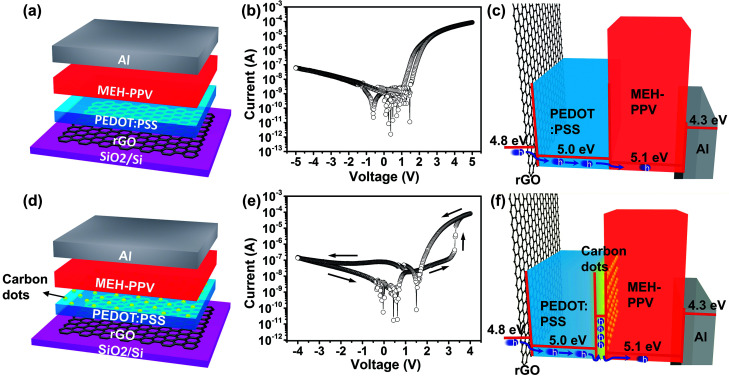
(a) Schematic structure, (b) typical *I*–*V* characteristics and (c) energy band diagram of the rectifying device. (d) Schematic structure, (e) typical *I*–*V* characteristics (the arrows represent the sweep directions) and (f) energy band diagram of the rectifying memory device.

To obtain the self-rectifying memory effect, carbon dots are introduced as the memory active layer due to their carrier trapping behavior within polymers (Fig. 2(d)). Carbon dots were prepared by pyrolyzing citric acid as described elsewhere.^33^ The UV-vis absorption and PL spectra of carbon dot are demonstrated in Fig. 3(a), from which an apparent UV-vis absorption band centered at 335 nm is observed and the maximum PL emission occurs at a wavelength of 445 nm, suggesting their semiconductor properties. High quality TEM image shows that carbon dots have a diameter of ∼4 nm (Fig. 3(b)), suggesting their strong ability to capture charges. Moreover, current–voltage (*I*–*V*) characteristics of the self-rectifying memory device are investigated and demonstrated in Fig. 2(e). When a negative bias was initially applied to the rGO bottom electrode, the device exhibited a high resistance state (HRS, namely OFF state). By applying a low positive voltage, the holes injected from rGO electrode were captured by carbon dots, the carbon dots served as trap centers due to the boundary and quantum confinement effect.^34^ With the increase of sweep voltage, the injected carriers increase rapidly and the traps were nearly filled. When the power supply approaches the threshold voltage, the traps were filled completely, the device underwent a resistive switching from HRS to low resistance state (LRS, namely ON state). The device maintained a LRS with trap filling when the voltage swept from 4 to 0 V. The device exhibits rectifying effect at LRS due to the Schottky effect coming from the bilayer's energy level offset with electrodes. When the power was turned off, the captured charges might be released from the trap centers due to the shallow traps of carbon dots and the device returned to HRS.

**Fig. 3:**
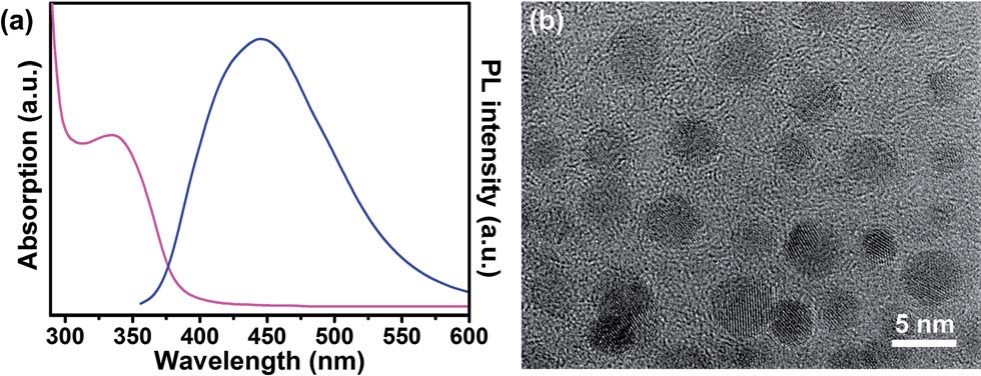
(a) UV-vis absorption and PL spectra of the carbon dots. (b) TEM image of the carbon dots.

The performance of the self-rectifying memory devices was evaluated under ambient conditions. Fig. 4(a) shows the typical *I*–*V* characteristics of the self-rectifying memory device under positive voltage sweep. The device could not maintain in the ON state steadily and it relaxed to the OFF state as once the power was removed, suggesting its volatile property. Fig. 4(b) shows the statistical distribution of ON-/OFF-state current (measured at 3 V) of the operative memory cells. The distribution of OFF- and ON-state current values lays within two orders of magnitude, the maximum currents at ON state and OFF state are about 10^−4^ and 10^−7^ A, respectively, with a ON/OFF current ratio of 10^2^ to 10^3^ : 1, reducing the misreading probability during the operation process. We also measured 40 randomly selected memory cells to evaluate the uniform distribution of switching threshold voltages, as shown in Fig. 2(c). The memory cells demonstrate an average value nearly 3 V of switching voltage. The retention time of the ON and OFF state with a continuous 3 V is measured in Fig. 4(d). The ON state can be maintained by applying a refreshing pulse of 4 V every 5 s, a slightly degradation was observed at the beginning and underwent stable with an ON/OFF current ratio of 30. The curves of first sweep in Fig. 4(e) and (f) indicated that both devices with the same structure basing on rGO electrode and ITO electrode can realize the self-rectifying memory effect, even though the former one exhibits higher endurance stability. After 100 consecutive voltage sweeps, the rectifying ratio of rGO/PEDOT : PSS/carbon dots/MEH-PPV/Al device still maintains 10^2^ (Fig. 4(e)). Simultaneously, the ON/OFF ratio of the device at 3 V remained approximately 36. In contrast, the phenomenon of rectifier and memory gradually disappeared after six consecutive voltage sweeps for ITO based devices (Fig. 4(f)). This performance degradation was caused by the unstable property of the ITO/PEDOT : PSS interface in ambient environment. The acidic PEODT : PSS solution can etch ITO during the polymer spin-coating process, and hydrolysis of deposited PEDOT : PSS *via* moisture absorption can also etch ITO.^35^ On the contrary, rGO electrodes are physically, chemically and electrically stable in ambient environment, guaranteeing a higher endurance stability of self-rectifying memory devices with rGO electrode.

**Fig. 4:**
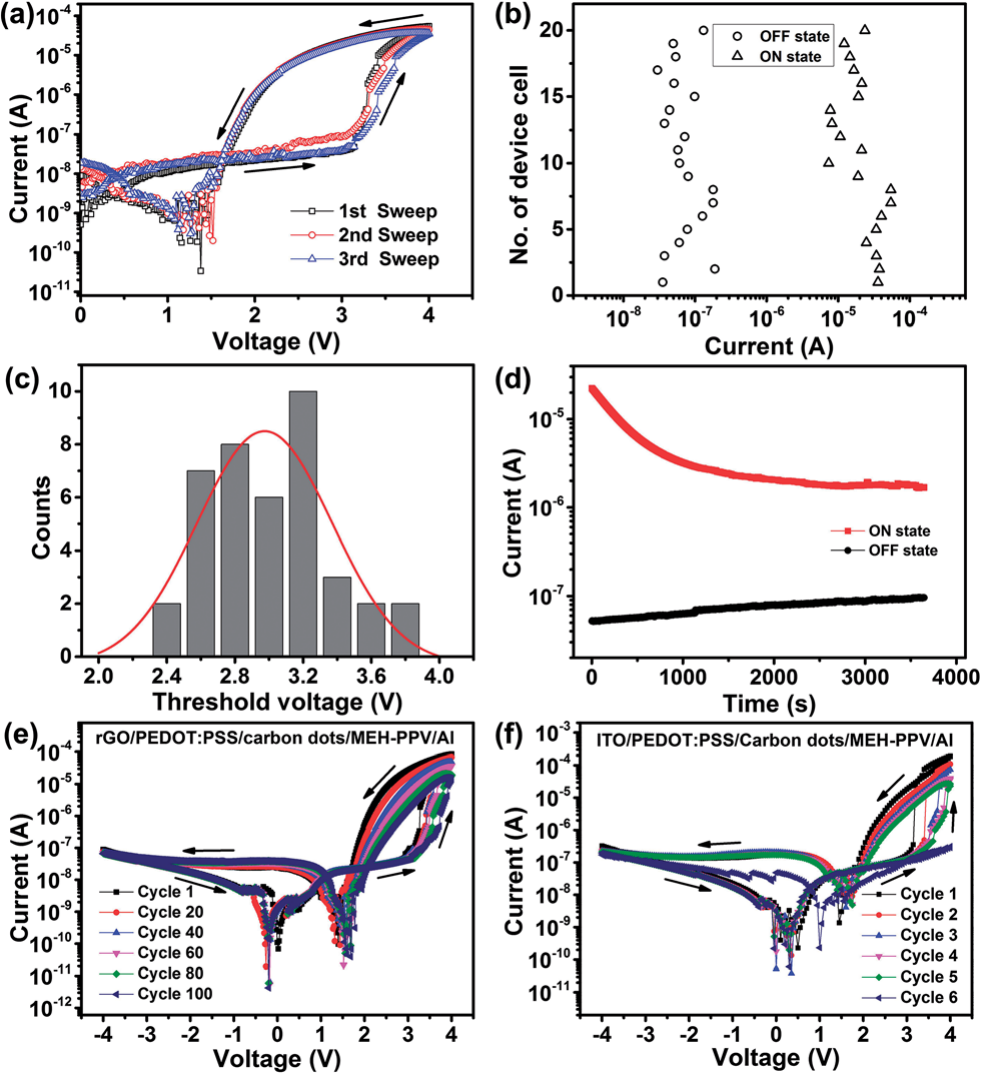
(a) Typical *I*–*V* curves of the rGO/PEDOT : PSS/carbon dots/MEH-PPV/Al device. (b) Statistical distribution of the ON-/OFF-state currents measured at 3 V. (c) Statistics histograms of switching voltages of the rGO/PEDOT : PSS/carbon dots/MEH-PPV/Al devices from 40 memory cells. (d) Retention time for the rGO/PEDOT : PSS/carbon dots/MEH-PPV/Al device under a continuous positive bias stress. (e) Cycle endurance test of the rGO/PEDOT : PSS/carbon dots/MEH-PPV/Al device. (f) Cycle endurance test of the ITO/PEDOT : PSS/carbon dots/MEH-PPV/Al device. The arrows represent the sweep directions.

In summary, a self-rectifying polymer memory device with the configuration of rGO/PEDOT : PSS/carbon dots/MEH-PPV/Al has been designed and fabricated through solution process. The memory effect of the as-fabricated device is attributed to the carrier trapping effect of carbon dots within polymers and the corresponding rectifying characteristic comes from the bilayer's energy level offset with electrodes. The self-rectifying memory device exhibits a maximum rectification of 10^3^ in rectify model and a minimum ON/OFF current ratio of 121 in memory model. Moreover, the devices show high endurance stability of self-rectifying memory effect with rGO electrode compared to that with ITO electrode. Importantly, the solution process fabrication make this device extremely simple. Because of the self-rectifying memory feature, the simple devices have great potential application in cross-bar structure memory for high-density data storage.

## Conflicts of interest

There are no conflicts to declare.

## Supplementary Material
